# Pediatric Intussusception: Decreased Surgical Risk with Timely Transfer to a Children’s Hospital

**DOI:** 10.21767/2471-805X.100018

**Published:** 2016-10-08

**Authors:** Brian P Blackwood, Christina M Theodorou, Ferdynand Hebal, Catherine J Hunter M

**Affiliations:** 1 Division of Pediatric Surgery, Department of Surgery, Ann & Robert H. Lurie Children’s Hospital of Chicago, Chicago Ave, Box 63, Chicago; 2 Department of Pediatrics, Northwestern University Feinberg School of Medicine, 310 E. Superior St. Searle #4-490, Chicago; 3 Department of General Surgery, Rush University Medical Center, 1750 W. Harrison Street, Suite 785, Chicago

**Keywords:** Intussusception, Pediatrics, Transfer, Surgery, Risk

## Abstract

**Introduction:**

Intussusception is a potentially life-threatening condition, and a frequent cause of bowel obstruction during the first two years of life. We hypothesized that patients who were transferred from outside community hospitals, or OSH, without tertiary care capabilities for pediatric services to a large academic children’s hospital with intussusception were more likely to require operative management for their intussusception than those who were directly admitted.

**Methods:**

The electronic medical record was queried for patients presenting to Ann and Robert H. Lurie Children’s Hospital of Chicago with a diagnosis of intussusception (July 1^st^, 2009–July 1^st^, 2014). Age, sex, symptom duration, radiologic management, and surgical care were recorded. OSH and transfer reports were analyzed for those patients that presented as a transfer. Statistical analysis was performed.

**Results:**

We identified 270 patients with intussusception. 232 (80%) were successfully treated non-surgically. 58 (20%) required surgical management. Of the patients requiring surgery, there were 38 reductions (24 laparoscopic, 14 open) and 20 bowel resections (1 laparoscopic, 19 open). Of those patients requiring surgery, 37 (63.8%) had presented as a transfer from an OSH. We found that transferred patients, requiring surgery, spent a mean 7.77 hours at the OSH compared to 4.03 hours for the transferred patients that did not require surgery (p=0.0188). There was no significant difference in transport time (p=0.44).

**Conclusion:**

In conclusion, we identified the amount of time patients spend at hospitals without pediatric surgical capabilities as an independent risk factor necessitating surgical management of intussusception. These data suggest that patients with intussusception who present to hospitals without pediatric radiology or pediatric surgery, should be transferred in an expedited fashion. In the event of a failed enema reduction at an OSH, the transport of the patient should not be delayed as this may result in a higher likelihood of surgical management.

## Introduction

Intussusception is a potentially life-threatening cause of bowel obstruction that commonly occurs in children. Most cases are able to be successfully treated with enema reduction, with success rates of up to 91% [1]. Studies have looked at risk factors for pediatric patients requiring surgical intervention for intussusception and have found that initial failure of reduction, existence of a lead point, bowel wall thickening on ultrasound, and longer length of symptoms prior to presentation all result in an increased likelihood of surgery [2]. Several of these factors may be associated with bowel ischemia which may further contribute to difficulty with reduction.

Recent work has indicated that children who present to Children’s Hospitals are less likely to require an operation for intussusception as compared to those that present to a Non-Children’s Hospital [3]. In this study we hypothesized that patients with intussusception who presented as transfers from outside community hospitals [4] to a large academic children’s hospital were more likely to require operative management for their intussusception, than those who presented directly to a large academic children’s hospital.

Herein we show that children initially presenting to OSH without tertiary care capabilities for pediatric services are more likely to require operative management of their intussusception than children who initially present to a children’s hospital. Additionally, a longer time spent at OSH prior to transfer was independently associated with higher rates of operative management.

## Methods

After obtaining IRB approval, a single center retrospective review was performed at a free-standing major metropolitan children’s hospital. The electronic medical record was queried for all pediatric patients presenting with a diagnosis of intussusception, either as a transfer or as a primary admission, during the time period from January of 2009 through January of 2014.

Patients were included if they had either abdominal ultrasound (U/S) or computed tomography [5] evidence of the most common type of pediatric intussusception, an ileocolic intussusception. Patients were excluded if they did not have radiologic evidence to support the diagnosis of ileocolic intussusception.

Demographics including age, sex, symptom duration, diagnostic imaging, radiologic management, and surgical care were recorded. Further analysis was done for those patients that presented as a transfers from OSH that did not have tertiary care capabilities for pediatric services. For these patients, OSH and transfer reports were analyzed. OSH admission length of time, and transfer time were recorded. OSH admission time was measured from the recorded admission time to the time at which transfer was decided on. The time at which the transfer was decided on was indicated by the time at which a call was made to our institution requesting a transfer. Transfer time was then measured from the time that the transfer team was first contacted to the time that the patient arrived at our institution.

Graphs and tables were generated using Excel. Statistical analysis was completed with Students T-Test or ANOVA where applicable using Graph Pad Prism 6 Software. Differences were considered significant at p<0.05.

## Results

Over the five-year period investigated, 356 individual encounters were identified with patients having the diagnosis of an intussusception. Of the initial 356 encounters identified, there were a total of 270 patients that met the inclusion criteria, making up 290 individual encounters with imaging-confirmed intussusception. The 66 excluded encounters were due to a lack of radiologic evidence of intussusception. The majority of the patients, 184 (63.4%), were male and 106 (36.6%) were female. The patients’ ages ranged from 6 weeks old to 15 years old and there was an average age at presentation of 1.93 years old. Only 5 patients over the age of 10 were identified. All five patients presented initially to our institution and required an operation. The average age of the non-operative patients was 1.88 years and the average age of the operative patients was 1.95 years. There was no significant difference in age when operative patients were compared to non-operative patients (p=0.8107). There were a total of 20 (6.8%) recurrences. There were 170 (58.7%) patients who presented initially to our institution and 120 patients (41.3%) who presented as transfers from an OSH.

The large majority of patients, 232 (80%), were treated with non-operative management (radiologic reduction or observation) and 58 (20%) required surgical intervention. However, further analysis revealed a non-operative success rate of 87.6% (149/170 patients) for those patients who presented initially to our institution and a success rate of only 69.1% (83/120 patients) in those patients who presented as transfers from OSH. Of the total patients requiring surgery, there were 38 reductions (24 laparoscopic, 14 open) and 20 bowel resections (1 laparoscopic, 19 open).

**Figure 1:**
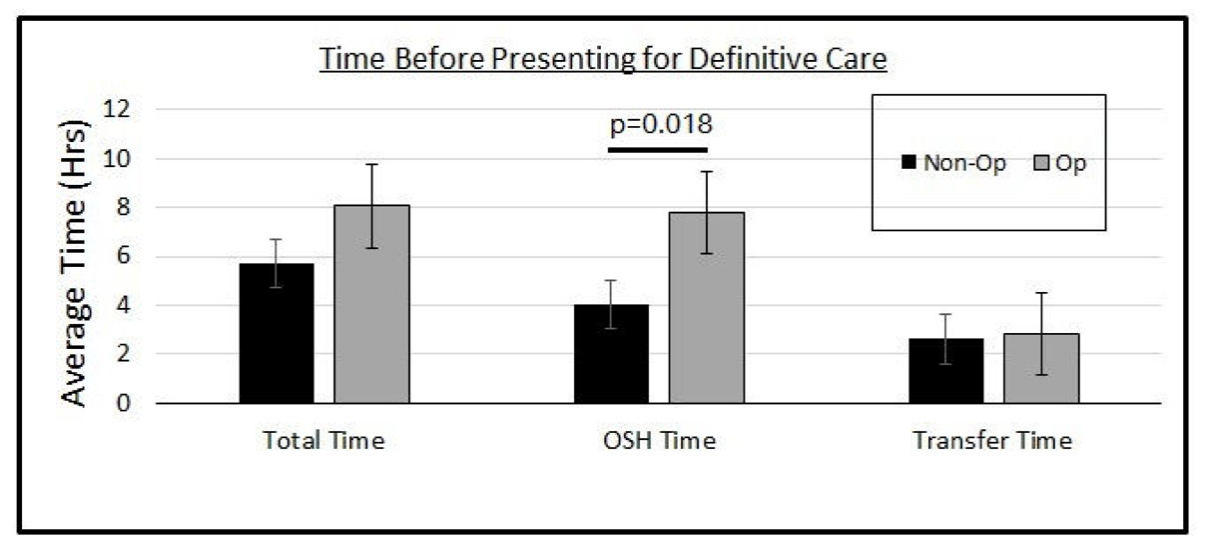
Analysis of those patients that presented as OSH transfers revealed that those that required operative intervention spent a significantly longer time at the OSH (p=0.018). There was no significant difference in transport time.

Of those 58 patients requiring surgery, the majority, 37 (63.8%), had presented as a transfer from an OSH. Looking specifically at the patients that presented as a transfer, there was no statistical difference in age or gender between the group of patients that had non-operative management versus operative management (p=0.764, p=0.8107 respectively). There was also no difference in the duration of symptoms (p=0.689) (Table 1). However, we did find that transferred patients requiring surgery spent a mean of 7.77 hours at the OSH compared to a mean of 4.03 hours for transferred patients that did not require surgery (p=0.0188). There was no significant difference in transport time between these two groups (p=0.44) (Figure 1).

## Discussion

Intussusception remains a potentially life-threatening condition in the pediatric patient, yet the majority of patients can avoid an operation and fully recover when treated by skilled pediatric radiologists and pediatric surgeons. Multiple attempts at reduction are sometimes required; however intussusceptions are successfully treated with therapeutic enemas in 75% to 91% of cases [1]. Previous studies have looked at risk factors for pediatric patients requiring surgical intervention for intussusception. Specific risk factors identified for requiring surgery have been failure of reduction, existence of a lead point, bowel wall thickening on ultrasound, and longer length of symptoms prior to presentation [2].

Since the 1970s contrast enema has been the primary intervention for intussusception with high rates of success [6]. It is therefore important to identify reasons that reduction with enema might fail. Studies looking at predictors of failed enema reduction found that children with symptoms greater than 24 hours, bloody diarrhea, lethargy, and further distal extent of the intussusception were more likely to require surgery [7].

The type of radiographic enema reduction, pneumatic versus contrast dye, has also been noted to affect outcome. There has been a trend at academic centers towards performing primarily pneumatic reductions, and evidence exists to suggest that pneumatic enema is superior to the liquid contrast enema [8–14]. Some believe this to be a result of an increased intraluminal pressure that is obtained with the air enema [15]. In our study only 20 pneumatic enemas were performed, with the remainder being contrast enemas, and still there was an 80% rate of successful non-operative management. With only 20 pneumatic reductions in our series we cannot adequately compare the two techniques to draw a definite conclusion, but they appear to have similar success rates.

Others have looked at the effect of hospital size on the success of non-operative management. These studies found that larger children’s hospitals have better rates of successful enema reduction as compared to hospitals with less experience with pediatric cases [16]. This is likely related to the larger number of cases of pediatric intussusceptions treated in a large tertiary care center allowing for more proficiency in the radiologists performing the enema reduction. A reported rate of 55% of patients requiring operative management at large children’s hospitals as compared to a rate of 68% at a non-children’s hospital has been described [3]. This may be due to a larger volume of cases and expertise in the medical management of intussusception at large children’s hospitals.

In our study we found that the majority (63.8%) of patients requiring operative intervention did not present directly to our institution, instead these patients arrived in a delayed fashion as transfers from OSH. This finding prompted a sub-analysis of those patients that were presenting as transfers. Interestingly, patients treated at children’s hospitals for intussusception have been found to be more likely to present as transfers from an outside hospital and those transferred patients were more likely to have sepsis, be acidotic, and require an operation [17]. However, this study did not determine the cause of these differences. We did not have patients presenting as transfers who were septic, but looking specifically at the subset of transferred patients in our study, we found that those patients that went on to require surgery spent a significantly longer period of time at the OSH than those patients that did not require surgical intervention. On average the patients that required surgery spent 3.74 hours longer at the OSH than the patients that were successfully managed non-operatively. Another study found that patients were also found to have an increased risk of requiring surgery when they presented initially to non-academic hospital [18]. This study shows an increased risk of operative intervention when these patients spent longer than 24 hours at the non-academic hospital, but our data demonstrates the window for definitive therapy may be much smaller.

Our data indicates that in pediatric patients presenting with intussusception, delays in definitive therapy from skilled pediatric radiologists and pediatric surgeons leads to an increased risk of operative management. There is evidence showing that radiologic reduction is more likely to fail the longer the duration of symptoms before intervention is attempted [2,19,20]. This could be the reason why these transferred patients are presenting and requiring operative intervention. While many pediatric problems may be treated adequately at OSH, it is evident that a higher degree of training and specialization in pediatric radiology and pediatric surgery does make a difference in this subset of patients.

One limitation of our study occurs in the data from the OSH itself. Unfortunately, the data we had access to for our analysis was limited to what was provided in the patients’ transfer reports. Therefore, we had limited information as to which patients had undergone attempts at radiologic reduction at the OSH and which of those were successful or not. Failed attempts at reduction at the OSH would definitely contribute to the delay in presentation and those patients would be more likely as well to require surgery. However, our analysis of the patients who presented as transfers from OSH and went on to require surgery revealed no additional data or risk factors to indicate that they would have required surgery regardless of what type of hospital they initially presented to. Even without these OSH data, our analysis highlights a potentially modifiable risk factor for intussusception patients going on to require surgical intervention.

## Conclusion

Based on our experience intussusception that presents directly to our institution can be managed non-operatively 87.6% of the time. This success rate significantly declines to a rate of 69.1% when patients arrive as OSH transfers. We have identified the amount of time patients spend at hospitals without pediatric surgical capabilities as an independent risk factor necessitating surgical management of intussusception. These data suggest that patients with the diagnosis of intussusception who present to hospitals without tertiary care capabilities for pediatric services should be transferred in an expedited fashion.

## Figures and Tables

**Table 1:** Retrospective review revealed that 63.8% of patients requiring surgery for intussusception had presented as a transfer from an OSH. There was no significant difference in age, sex, or duration of symptoms.

Patients Transferred for Intussusception			
Patient Characteristics	Non-Operative	Operative	P-Value
Number (N)	83 (69.2%)	37 (30.8%)	P<0.0001
Mean Age (Years)	1.88	1.95	P=0.810
Gender			
Male	56 (67.5%)	24 (64.9%)	P=0.764
Female	27 (32.5%)	13 (35.1%)	
Mean Duration of Symptoms (days)	1.9	2.1	P=0.689
